# Hydroelastomers: soft, tough, highly swelling composites†

**DOI:** 10.1039/d2sm00946c

**Published:** 2022-09-06

**Authors:** Simon Moser, Yanxia Feng, Oncay Yasa, Stefanie Heyden, Michael Kessler, Esther Amstad, Eric R. Dufresne, Robert K. Katzschmann, Robert W. Style

**Affiliations:** Department of Materials, ETH Zürich Switzerland robert.style@mat.ethz.ch; Department of Mechanical and Process Engineering, ETH Zürich Switzerland rkk@ethz.ch; Institute of Materials, EPFL Switzerland

## Abstract

Inspired by the cellular design of plant tissue, we present an approach to make versatile, tough, highly water-swelling composites. We embed highly swelling hydrogel particles inside tough, water-permeable, elastomeric matrices. The resulting composites, which we call hydroelastomers, combine the properties of their parent phases. From their hydrogel component, the composites inherit the ability to highly swell in water. From the elastomeric component, the composites inherit excellent stretchability and fracture toughness, while showing little softening as they swell. Indeed, the fracture properties of the composite match those of the best-performing, tough hydrogels, exhibiting fracture energies of up to 10 kJ m^−2^. Our composites are straightforward to fabricate, based on widely-available materials, and can easily be molded or extruded to form shapes with complex swelling geometries. Furthermore, there is a large design space available for making hydroelastomers, since one can use any hydrogel as the dispersed phase in the composite, including hydrogels with stimuli-responsiveness. These features make hydroelastomers excellent candidates for use in soft robotics and swelling-based actuation, or as shape-morphing materials, while also being useful as hydrogel replacements in other fields.

## Abstract 2

Inspired by the cellular design of plant tissue, we present an approach to make versatile, tough, highly water-swelling composites.

## Graphical abstract

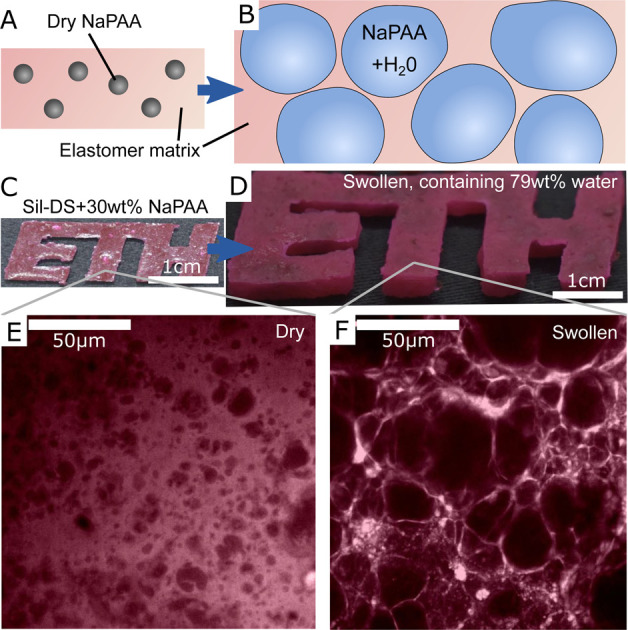

## 1 Introduction

Hydrogels are probably one of the most important classes of soft material due to their central role as a main component of living tissues. The high water content of synthetic hydrogels commonly makes them biocompatible,^1^ and allows them to host biochemical reactions.^2^ Hydrogels can swell in volume by absorbing hundreds of times their weight in water, allowing actuation and large changes in structure. Furthermore, they can be made stimuli-responsive: capable of changing material properties or degrading in response to external stimuli like light, pH, temperature, or electric fields.^3^ Despite this, it is interesting that there are still only a relatively modest number of industrial applications of hydrogels outside of the food industry. By volume, super-absorbent polymers in diapers and agriculture are the dominant applications.^4^ Emerging biomedical applications also include tissue engineering,^1^ wound healing^2^ and drug delivery.^5^

The limited adoption of hydrogels is, in large part, due to various drawbacks of common, bulk-produced hydrogels. For example, simple synthetic hydrogels typically have low stretchability, are brittle,^6^ and are not straightforward to fabricate as they rely on free-radical polymerization, which is oxygen sensitive.^7^ Hydrogels can also be tricky to adhere to most surfaces,^8^ and will normally dehydrate in air.^9^ In the latter case, the loss of water can cause changes to the mechanical properties of a hydrogel by multiple orders of magnitudes, as hydrogels typically dry to become tough, stiff, glassy solids.^10^ Recently, novel, specialized hydrogels have been developed that can overcome many of these shortcomings (*e.g.* ref. 6, 9, 11, 12, 13, 14, 15, 16 and 17). However, even with these advances, hydrogels have not yet been able to replace materials like silicones and polyurethanes in most commercial applications requiring soft materials. Silicone and polyurethane elastomers are popular, as they combine many useful properties in a single material. They are naturally stretchable, tough, and keep their mechanical properties across a wide range of chemical and thermal environments.^18^ They also easily adhere to a range of surfaces, and can be simply formed into any desired shape simply by mixing two liquid precursors. Furthermore, these properties can be combined with mold/mildew resistance, chemical inertness, UV resistance, and the ability to be dyed with non-leaching color.

Here, we demonstrate how hydrogel/elastomer composites can combine desirable properties of hydrogels and elastomers. These hydroelastomers consist of microscopic hydrogel particles embedded in elastomeric matrices. Our material design is motivated by nature's strategy of combining highly swellable osmotic inclusions (cells) inside tough matrices to produce robust materials like plant tissue.^19^ Our strategy also builds on previous work showing that liquid inclusions can enhance fracture and stiffness properties of elastomeric materials.^19–22^ Our composites inherit the water swelling characteristics of their hydrogel components, while inheriting properties such as outstanding toughness, stretchability, and ease of fabrication of their elastomeric component. Indeed, the materials are highly processable, as they can even be 3d printed into complex geometries with non-uniform structures. Hence, we envisage that hydroelastomers should have a range of potential uses replacing hydrogels or other soft materials in applications including soft robotics,^23–25^ shape-morphing materials,^26^ swelling sealants,^27,28^ and water-retention in agriculture.^29^

## 2 Material creation

**Fig. 1:**
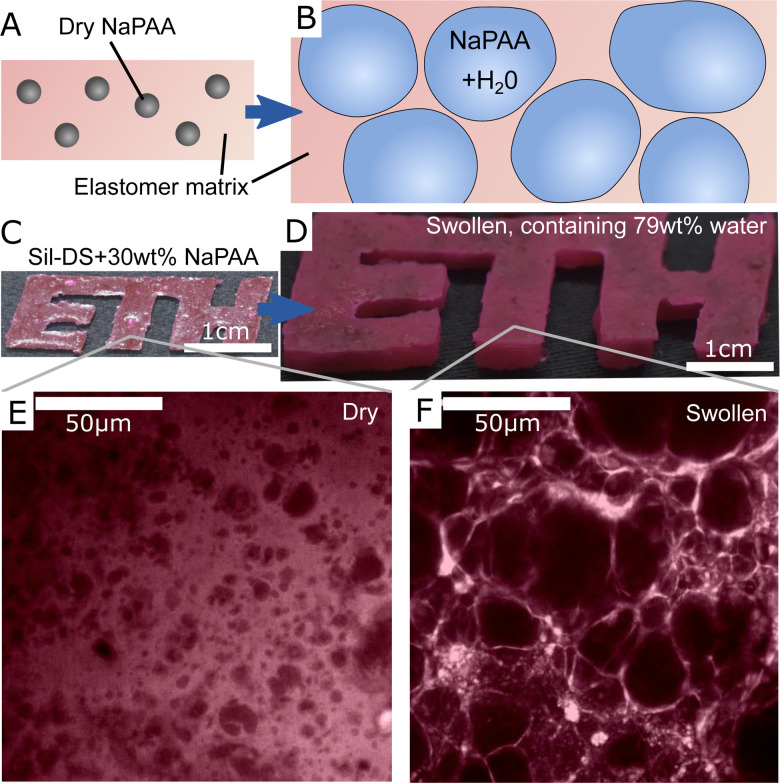
An overview of hydroelastomers. (A and B) show the swelling principle of the material, whereby highly swelling microgels are embedded in tough, permeable elastomers to make tough, swellable composites. (C and D) show a sample made of 30% sodium polyacrylate (NaPAA) in silicone (Sil-DS) before and after swelling in water. The color comes from rhodamine B dye. (E and F) Confocal microscopy images showing the microstructure of the samples in C and D. Dark areas are the hydrogel.

To create our composites, we synthesize dry, cross-linked sodium polyacrylate (NaPAA) microgels, embed them in a range of soft elastomers, and then swell the resulting composites in water (Fig. 1A and B). In principle, we could use any of the wide range of hydrogels as microgels for this purpose. However, NaPAA is convenient as it is a common, commercial, super-absorbent polymer, capable of absorbing hundreds of times its weight in water. We fabricate dry NaPAA powder *via* emulsion polymerization, to produce particles with an approximate diameter of 30 μm when fully swollen in water (see Materials and methods and ESI† for further details). For the elastomeric matrix, we use common, commercial elastomers: Smooth-on Dragonskin 30 (Sil-DS), a relatively stiff, tough silicone, with a measured Young's modulus, *E* = 1.12 MPa; Smooth-on Ecoflex 10 (Sil-EC), a much softer silicone, with *E* = 33 kPa; and Smooth-on Vytaflex 40 (PU-VY) a polyurethane, with *E* = 1.4 MPa. These elastomers are generally considered as non-swelling in water, but are permeable to water transport *via* molecular diffusion.^30,31^

We note that almost any water-permeable elastomer or gel could be used as a matrix material. Indeed, one could use tough hydrogels – which would result in materials that are conceptually similar to microgel-reinforced hydrogels.^32,33^ The only constraint on the matrix is that it must be soft enough to be deformed by the osmotic pressure, *Π*, generated in the swelling microgels. As shown in the ESI,† using data from ref. 34, *Π* can reach up to almost 100 MPa. Dry microgels embedded in a matrix will swell until their osmotic pressure reaches a value comparable to *E*.^35^ Thus, if *E* ≳ 100 MPa, microgels will not significantly swell from their dry state. On the other hand, we expect microgels to swell fairly freely inside matrix materials with *E* ≲ 10 MPa, which will result in a high water content of the fully swollen composite.

The final fabrication step involves mixing dry NaPAA powder with the liquid precursors of the matrix material with an centrifugal mixer. The mixture is then degassed and cured overnight in an oven at 40 °C. We use a mass fraction, *ϕ*_p_, of dry microgel in the as-prepared composite in the range of 10–30 wt%. It is difficult to work with higher fractions without trapping air bubbles, which compromise the material properties. This problem chiefly arises when working with matrix precursors having large viscosity.

A typical example of a composite made from 30 wt% of dry NaPAA in Sil-DS is shown in Fig. 1C and D. After swelling in de-ionized water for 14 days, the mass of the sample increases by 275% until it reaches an equilibrium swelling that is almost 80 wt% water. The microgels expand significantly upon swelling, ultimately stretching the water-permeable matrix to form thin walls between the individual inclusions (Fig. 1E and F). It is likely that there is very little adhesion between the microgels and the matrix, due to their respective hydrophilicity/hydrophobicity. However the swelling presses the microgels into the surrounding matrix, ensuring constant contact between these two phases.

## 3 Stiffness

**Fig. 2:**
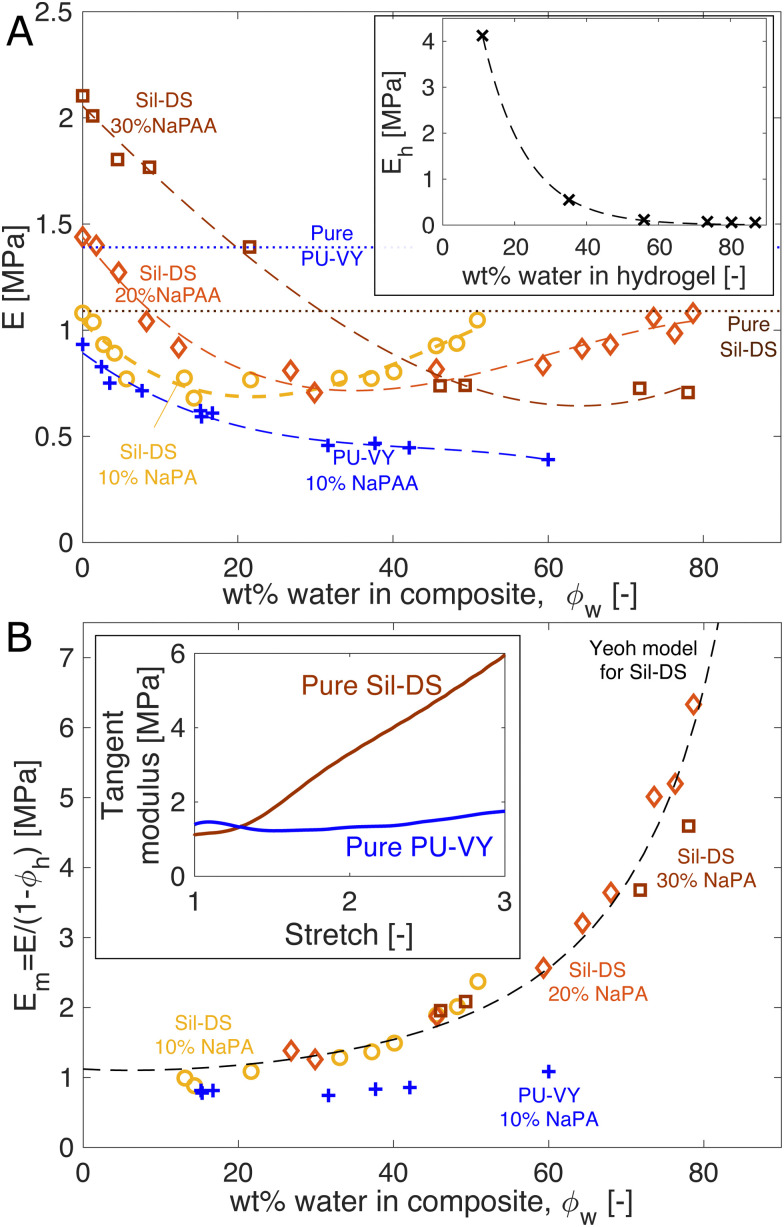
Composite stiffness is a surprisingly weak function of swelling. (A) Composite stiffness *versus* water content. The yellow, orange, and brown data sets are Sil-DS composites with 10, 20, and 30 wt% of dry NaPAA, respectively. The blue data set is a PU-VY composite with 10 wt% of dry NaPAA. The dashed curves are best-fit cubic polynomials, while the dotted lines show *E* for pure Sil-DS and PU-VY. Inset: The stiffness of the NaPAA (measured in bulk samples) as a function of its water content. The dashed curve is a best-fit exponential function. (B) The effective modulus of the matrix *versus* swelling. We use only data points from A, where the microgels contain more than 50 wt% water, to give *E*_m_ = *E*/(1 − *ϕ*_h_). The dashed line is our theoretical prediction of *E*_m_ for Sil-DS with 10 wt% of dry NaPAA. The inset shows the tangent moduli of pure, dry Sil-DS and pure, dry PU-VY when stretched uniaxially. These moduli are qualitatively similar to the composite stiffness results in (B).

We study the mechanical properties of the composites as a function of their water content. Fig. 2A shows the Young's modulus of pure NaPAA hydrogel (inset), and silicone and polyurethane composites as a function of the total mass fraction of water in the samples, *ϕ*_w_. The Young's modulus is measured *via* uniaxial tensile testing. The silicone composites are initially formulated with 10, 20, and 30 wt% of dry NaPAA, while all polyurethane composites are made with 10 wt% of dry NaPAA. The dry silicone composites are stiffer than their respective pure matrix materials, while the polyurethane composites are softer. Upon initial swelling, all the samples start to soften. However, interestingly, the silicone-based ones subsequently stiffen upon further swelling – especially the 10 and 20 wt% samples.

We can understand the qualitative changes in stiffness during swelling in terms of the properties of the microgels and the surrounding matrix. When dry, NaPAA is a glassy solid that is orders of magnitude stiffer than the surrounding matrix, and thus would be expected to stiffen the composite. This probably explains why the dry, higher NaPAA-content silicone composites are initially stiffer than pure silicone (in contrast, residual water in the ‘dry’ NaPAA powder probably hinders polymerization of the polyurethane samples, reducing their stiffness). As the composites swell, their stiffness is determined by a competition between softening of the microgels and stretching of the matrix. The microgel softening occurs rapidly as it swells, as shown by the inset in Fig. 2A – in a manner chararacteristic of the swelling of many hydrogels. This softening is likely responsible for the initial decrease in stiffness of all the composites upon swelling. By contrast, changes in composite stiffness at higher swellings are probably controlled by stretching of the matrix phase. This conclusion is supported by the data in the inset of Fig. 2A, which shows that microgels with a water content of >50% have a stiffness that is essentially negligible in comparison to that of the composite.

We gain further insight into the origin of the large-swelling behavior by isolating the contribution of the matrix for the data in Fig. 2A. The law of mixtures estimates composite stiffness as *E* = *E*_m_(1 − *φ*_h_) + *E*_h_*φ*_h_, where *E*_m_ is the effective modulus of the matrix, *E*_h_ is the microgel stiffness (the subscript h stands for hydrogel), and *φ*_h_ is the volume fraction of swollen microgels in the composite. For the highly swollen composites, where *E*_h_ ≈ 0, we can estimate *E*_m_ = *E*/(1 − *ϕ*_h_) (we assume *φ*_h_ = *ϕ*_h_, the mass fraction of hydrogel). Plotting *E*_m_ for the samples where the microgels are more than 50% water collapses the silicone data nicely (Fig. 2B). This analysis highlights that the silicone matrix appears to stiffen dramatically as it is stretched by swelling – up to 6 times its original modulus. By contrast, the polyurethane composite shows no real evidence of strain stiffening. This observation is consistent with the strain-stiffening behavior of the pure matrix materials (shown for uniaxial tension in the inset of Fig. 2B).

Indeed, we can use the measured nonlinear properties of the matrix materials to predict *E*_m_ with good accuracy. We fit the results of a uniaxial tension experiment on a pure Sil-DS sample with a hyperelastic Yeoh model. Then, we create a model of the composite as a sphere of this matrix material containing a growing spherical cavity. We inflate the cavity step-wise. At each step, we calculate the average incremental modulus of the matrix in response to a unidirectional stretch, while holding the cavity volume fixed. The results for a silicone composite with 10 wt% of dry NaPAA are given as the dashed curve in Fig. 2B (further curves for higher NaPAA loadings, and details of the calculation are given in the ESI†). The model captures the trend shown by the data, suggesting that the increasing stiffness of the composite at high swelling is indeed caused by strain stiffening of the matrix.

## 4 Fracture energy

**Fig. 3:**
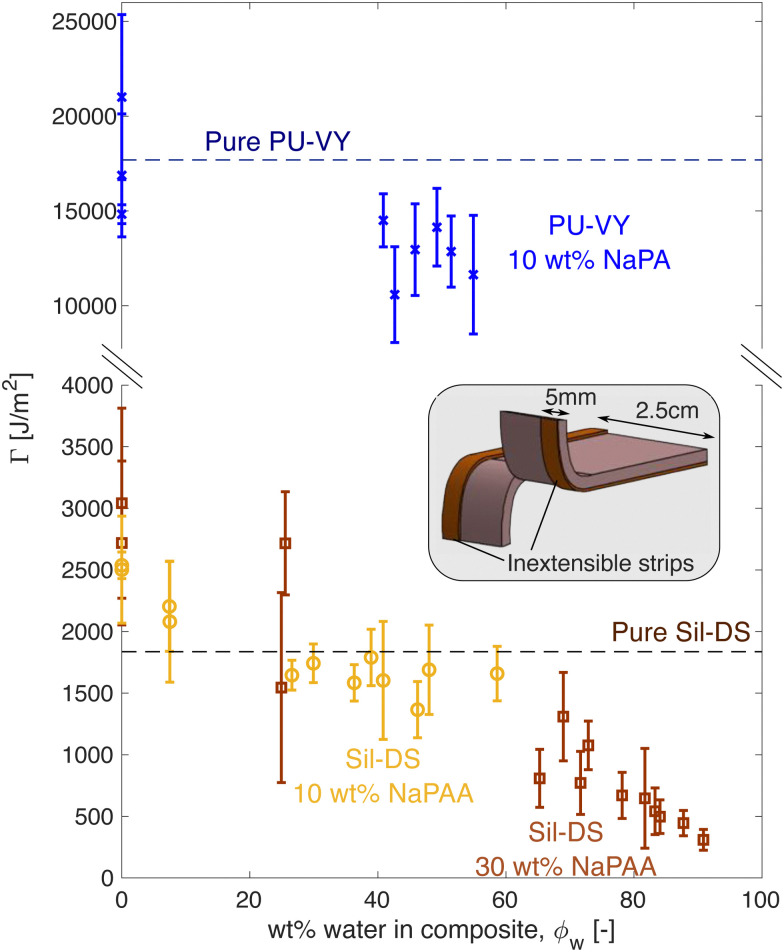
Fracture energy *versus* swelling for polyurethane (blue) and silicone (yellow/brown) composites. The inset shows a schematic of the test geometry used. Flexible, inextensible strips are glued to the sample edges to prevent leg stretching during the test. The sample thickness depends on the swelling state of the composite.

The composites inherit much of the toughness of their corresponding matrix material, even when highly swollen with water. Fig. 3 shows how fracture energy varies with swelling for different composites, measured *via* tear tests (*cf.* the schematic^36,37^). Interestingly, for dry composites, *Γ* is actually larger than that of the pure matrix material, especially for the silicone composites. However, this is not surprising, as it is known that stiff, glassy microparticles act to toughen silicones and polyurethanes.^38,39^ As the composites swell, *Γ* reduces. In particular, for the silicone composites, we see an almost linear decrease with *ϕ*_w_, approaching 0 as *ϕ*_w_ → 1.

The observed linear decrease in *Γ* with swelling conceptually fits with a simple law of mixtures approach: *Γ* = *Γ*_m_(1 − *ϕ*_h_) + *Γ*_h_*ϕ*_h_, where now *Γ*_m_ and *Γ*_h_ are the matrix and microgel fracture energies, respectively. The microgels should be very brittle, so *Γ*_h_ ≪ *Γ*_m_, and *Γ* ≈ *Γ*_m_(1 − *ϕ*_h_). When *Γ*_m_ is not a strong function of stretch, this expression yields a linear drop off in fracture energy with *ϕ*_h_, as seen in Fig. 3A. We note, however, that a stretch-independent value of *Γ*_m_ is rather unexpected, as recent experiments have shown that stretching silicone can have a significant effect on its fracture energy.^40^

## 5 Applications

**Fig. 4:**
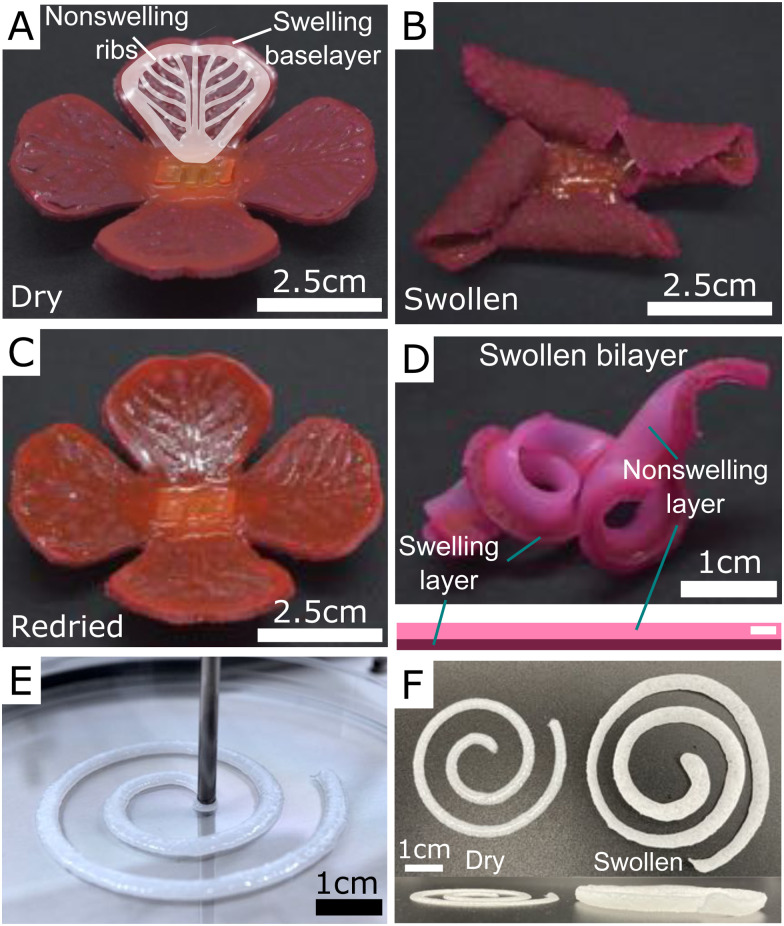
Using hydroelastomers to create complex swelling geometries. (A–C) A flower created with a 1 mm-thick swelling layer of 10 wt% NaPAA in Sil-DS, bonded to a 1 mm-thick rib structure, as shown in the schematic. The color comes from rhodamine B and Sudan I dye. After immersing in water, the petals curl up, before returning to the original shape when dried out. The slight color change is due to dye leaching during swelling. (D) A swollen bilayer consisting of a strip of non-swelling Sil-DS (dyed light pink) bonded to a strip of swellable composite (dyed dark pink). (E and F) The curing composite is shear thinning, allowing it to be printed *via* extrusion.

The simplicity of the fabrication process gives great flexibility in terms of creating objects with complex swelling characteristics. Fig. 4 shows a few simple demonstrations of using hydroelastomers as a swelling material. For example, Fig. 4A shows a flower where the ‘petals’ are made of a 1 mm-thick layer of swelling material (Sil-DS with 30 wt% of dry NaPAA) underlying another 1 mm-thick, non-swelling rib structure, made with pure Sil-DS (*cf.* schematic). We use a two-step molding process. When swollen in water, the petals curl upwards due to differential swelling, before repeatably returning to their original state when dried out. Similarly, Fig. 4B shows the results of swelling a simple, 15 cm-long bilayer consisting of a strip of swelling material (dark pink), bonded to non-swelling strip of pure Sil-DS (light pink). Upon swelling, the bilayer drastically changes its form by coiling up – just as in the process underlying the coiled shapes of vertebrate gut tubes.^41^ Videos of the flower and bilayer swelling are given in the ESI.† These videos illustrate the timescale for swelling of these materials.

Beyond the usage of molds, the composites can also be printed, as the curing microgel/polymer mixture has suitable rheological properties.^33^Fig. 4C shows a simple spiral of printed Sil-DS with 10 wt% NaPAA during printing and after swelling. In future, we anticipate that complex 3D shapes can be created by directly mixing particles and polymer in the printer to continuously tune particle concentration during printing. This mixing would allow the simple manufacture of complex swelling morphologies.

## 6 Discussion

**Fig. 5:**
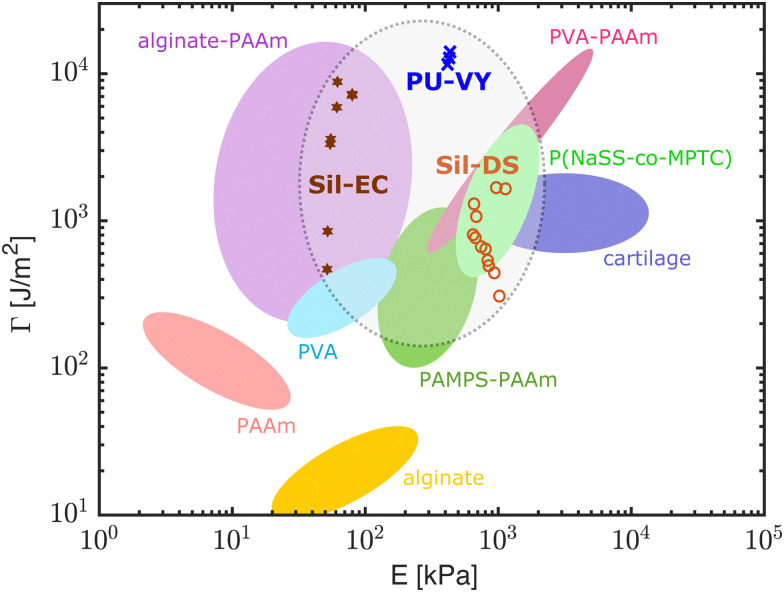
An Ashby diagram comparing our composites with state-of-the-art, water-swellable materials in terms of their fracture energy and stiffness. The diagram is modified from ref. 15. Out of our composites, only those with *ϕ*_w_ > 48% are shown here. The data from Fig. 2 and 3 are combined by using the best fit curves in Fig. 2 to calculate the corresponding *E* for each point in Fig. 3. PAAm: polyacrylamide, PVA: poly(vinyl alcohol), PAMPS: poly(2-acrylamido-2-methylpropanesulfonic acid), P(NaSS-*co*-MPTC): sodium *p*-styrenesulphonate-*co*-3-(methacryloylamino)propyl-trimethylammonium. Original references: PAAm, alginate, alginate-PAAm,^6^ PVA,^42^ PAMPS-PAAm,^11^ PVA-PAAm,^15^ P(NaSS-*co*-MPTC),^13^ cartilage.^43^

Our results show that our hydroelastomers are competitive when compared to other, tough, swelling materials. Fig. 5 shows an Ashby diagram giving the mechanical properties of a selection of state-of-the-art, water-swellable materials. Data points for our composites are shown for materials swollen with >48 wt% water. Our composites are in a similar range of stiffness to hydrogels, while having fracture properties that compare to state-of-the-art synthetic hydrogels. The polyurethane composites perform particularly well: matching, if not exceeding the best hydrogels available.

Our composites also have some significant advantages over the hydrogels in Fig. 5. For example, the mechanical properties of our composites are relatively fixed as they swell. This is very different to simple hydrogels, which dramatically stiffen – often going through a glass transition – as they dry out.^10^ We see this contrast directly in Fig. 2A, by comparing the change in stiffness of the composites (main figure) and pure NaPAA (inset) as they swell. Another advantage of our materials stems from the fact that the matrix materials used here, *i.e.* silicones and polyurethanes, are robust, commercial materials that can be used in harsh conditions. Silicones in particular have good heat resistance, chemical stability, and weatherability.^18^ We anticipate that these characteristics will carry over to the composites. Furthermore, hydroelastomers are simple to prepare. Once the microgels are created *via* emulsion polymerization, our composites are fabricated by mixing the ingredients together, briefly degassing, and then shaping with a mold, or *via* extrusion. In contrast, chemically crosslinked gels typically require oxygen-free conditions,^7^ polyvinyl alcohol gels require freeze–thaw^44^ or annealing and re-swelling,^15^ and polyampholyte and double network gels require dialysis or re-swelling steps.^13,45^

## 7 Conclusions

In conclusion, we present a simple, high water-content class of material that combines the useful properties of hydrogels and elastomers. The toughness and stretchability of these hydroelastomers match state-of-the-art tough hydrogels. Furthermore, they are easy to fabricate, and ideal for creating complex, 3D-printed, swelling materials. We anticipate hydroelastomers being of use in swelling-controlled actuation,^46^ and shape-morphing materials.^26^ The wide range of choices of matrix and inclusion materials gives great design flexibility in controlling composite properties. In particular, the material principle should allow us to simply combine hydrogel features like stimuli-responsiveness or high swelling capacity with elastomeric features like high toughness and stretchability. This strategy could be used to make tough composites that swell in response to triggers such as temperature, light, or electromagnetic fields.^3^ Currently, the main limitation on the swelling process is the speed of permeation of water through the matrix.^31^ However, this does aid in hindering dehydration – our samples can remain hydrated for weeks in normal ambient conditions. In the future, we anticipate substantially speeding up the swelling process by using matrix materials with higher water permeability, such as tough silicone hydrogels.^47,48^

### Materials and methods

We prepared dry NaPAA powder *via* emulsion polymerization. We synthesized sodium acrylate monomer by neutralizing acrylic acid (Sigma-Aldrich) with a 2.7 M solution of sodium hydroxide (Fisher Chemicals) in de-ionized water in a 1 : 1 molar ratio. To this, we added *N*,*N*′-methylenebis(2-propenamide) crosslinker (Sigma-Aldrich) as 4 wt% of the acrylic acid, Tween 80 surfactant (Sigma-Aldrich) as 0.47 vol% of the de-ionized water, and 2-hydroxy-2-methylpropiophenone initiator (Tokyo Chemical Industry) as 0.8 vol% of the de-ionized water. All chemicals were used as received. After stirring to ensure complete dissolution of all components, we formed an emulsion by adding 1 wt% of Span 80 surfactant (Sigma-Aldrich) in light mineral oil (Sigma-Aldrich) to the aqueous monomer solution in a ratio of 4 : 1. The mixture was vortexed and then polymerized with constant stirring for 7.5 minutes under a UV light with a nominal power of 15 W at a wavelength of 365 nm.

After polymerization, we washed and freeze-dried the resulting particles. This process involved centrifugation, followed by removal of the supernatant liquid, and subsequent re-dispersal of the particles in a solvent. The first two times, we used ethanol as the solvent. Subsequently, we repeated the washing eight times with a 1.5 M solution of NaCl (Fisher Chemicals) in de-ionized water. Finally, we flash-freezed the particles in liquid nitrogen, before freeze-drying at −80 °C and 0.2 mbar. This procedure resulted in a fine NaPAA powder.

**Fig. 6:**
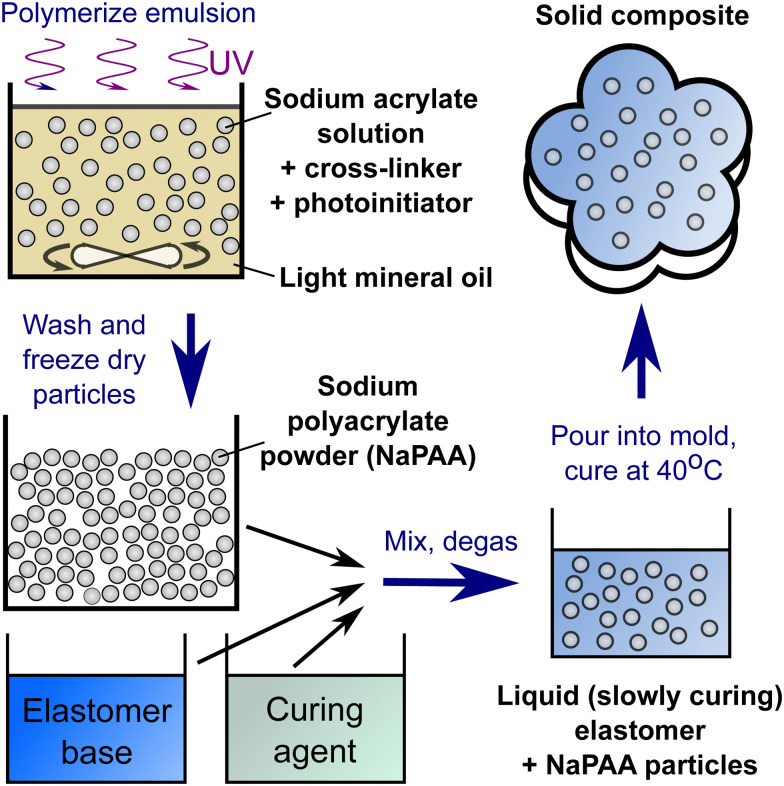
The basic procedure for creating hydroelastomers.

To prepare composite samples, we utilize two-component elastomer kits, which all have a pot life of *O*(10 min) at room temperature. These included Ecoflex 10 (Smooth-on), Dragonskin 30 (Smooth-on) and Vytaflex 40 (Smooth-on). All of these were mixed in the manufacturer-recommended ratio of base to hardener (*i.e.* a 1 : 1 mixture of parts A & B). The microgel powder was added to the curing matrix material, and mixed with a planetary centrifugal mixer (Flacktek Speedmixer) at 3600 rpm for one minute before being degassed in a vacuum chamber. Finally, the mixture was poured into a suitable mold before being cured at 40 °C. A summary of the procedure is shown in Fig. 6.

## Author contributions

S. M., O. Y., E. R. D., R. K. K. and R. W. S. designed and supervised the research. M. K. and E. A. developed the microgels and performed the 3D printing experiments. S. M. and Y. F. performed all other experiments. S. H. developed the theoretical model for stiffening. S. M., Y. F., R. K. K. and R. W. S. analyzed the data, S. M., O. Y., E. R. D., R. K. K. and R. W. S. wrote the paper.

## Conflicts of interest

There are no conflicts to declare.

## Supplementary Material

SM-018-D2SM00946C-s001

SM-018-D2SM00946C-s002

SM-018-D2SM00946C-s003

SM-018-D2SM00946C-s004
