# Supplementary material for: Hydroelastomers: soft, tough, highly swelling composites
Source: Soft Matter. 2022 Sep 6;18(37):7229–35. doi: 10.1039/d2sm00946c (PMC9516556; doi:10.1039/d2sm00946c)
Supplement: SM-018-D2SM00946C-s004 [file SM-018-D2SM00946C-s004.pdf]

Electronic Supplementary Material (ESI) for Soft Matter.
This journal is © The Royal Society of Chemistry 2022

Supplementary Information: 'Hydroelastomers: soft, tough, highly swelling composites'

Simon Moser,[1] Yanxia Feng,[1] Oncay Yasa,[2] Stefanie Heyden,[1] Michael Kessler,[3] Esther Amstad,[3] Eric R. Dufresne,[1] Robert K. Katzschmann,[2,*] and Robert W. Style[1,†]

[1]*Department of Materials, ETH Zürich, Switzerland*
[2]*Department of Mechanical and Process Engineering, ETH Zürich, Switzerland*
[3]*Institute of Materials, EPFL, Switzerland*

I. SAMPLE PREPARATION

We prepare the materials as described in the Materials and Methods of the main paper. We first fabricate NaPAA microgel particles via emulsion polymerization. Figure 1 shows images of the microgel particles in mineral oil (as fabricated), dried, and reswollen in water. Subsequently, we mix the particles with the two liquid components of the elastomer, degas the mixture, and pour this into a mold. After overnight curing, this yields the solid composite. The dry sample dimensions for the dog bone samples were 2.2 mm thick, 59.5 mm long and the central segment had a width of 10.8 mm. The dry trouser-test samples were 25 mm x 50 mm before notching and had an average thickness of roughly 0.7 mm. All dimensions were measured with calipers before testing.

An image of fully swollen Sil-DS composite with 10wt% of dry NaPAA is shown in Figure 2. This was taken with a confocal microscope (Nikon Ti2 microscope with a 3i Spinning Disk Confocal system).

II. SAMPLE CHARACTERIZATION

Samples were swollen in de-ionized water for different durations. We characterized the swelling by weighing the samples and comparing the results to their original dry mass to obtain the wt% of water in the samples, ϕ_w. This is related to the volume increase of the sample, relative to the dry volume (Figure 3).

We measured the elastic properties of samples by performing uniaxial tensile tests on thin samples with a tensile testing machine (TA.XTplus, Stable Microsystems). Samples were stretched at 0.05mm/s by 5% of their initial length, and their Young's modulus was calculated by fitting the linear section of the resulting stress/strain curve. At these slow strain rates, we saw no evidence of hysteresis during loading and unloading, so there is essentially no viscoelastic dissipation.

We measured sample fracture energy by performing trousers tear tests [1]. For these experiments, dry, rectangular specimens were fabricated. These were then swollen by different amounts before tear testing. To perform the experiment, thin, flexible, but inextensible stripes consisting of double-sided tape (Tesa 51970, with the protective layer removed) were superglued to the sides of the sample, as shown in Figure 3 of the main paper. These prevent stretching of the sample legs during tearing [2, 3], so that the fracture energy can simply be calculated $2F/b$, where F is the average force during crack propagation, and b is the sample thickness [1].

We measured the osmotic pressure versus swelling curve for NaPAA powder by vapor equilibration experiments (Figure 4). We placed a measured mass of powder in a chamber with an open container filled with magnesium chloride (MgCl) solutions with various osmotic pressures. The chamber also contained a continuously running fan to mix the air. The mass of the powder was recorded at regular intervals, and when this plateaued, we took this as the osmotic pressure of the hydrogel equalling the osmotic pressure of the solution. For osmotic pressures lower than 1 MPa, we directly measured the osmotic pressure of the MgCl solution with a freezing-point osmometer (Gonotec Osmomat 3000). Higher osmotic pressures were extrapolated from the literature [4].

III. QUALITATIVE IMAGES OF SAMPLES AT THE POINT OF FAILURE

The resistance to fracture of our composites is demonstrated in Figure 5A-C, which show examples of notch tests on composites with different matrix materials at different water contents (all originally made with 10wt% of dry NaPAA). In each image, the sample is at the point of crack propagation.

IV. VIDEOS

Two of supplementary videos show the swelling of the flower and the bilayer in Figure 4 of the main paper. In both cases, the dry samples are submerged into water and imaged at regular intervals. There is a slightly coloration of the water over time, due to dye (used for coloring purposes) leaching out of the samples. The final video shows the printing of the spiral in Figure 4 of the main paper.

* rkk@ethz.ch
† robert.style@mat.ethz.ch

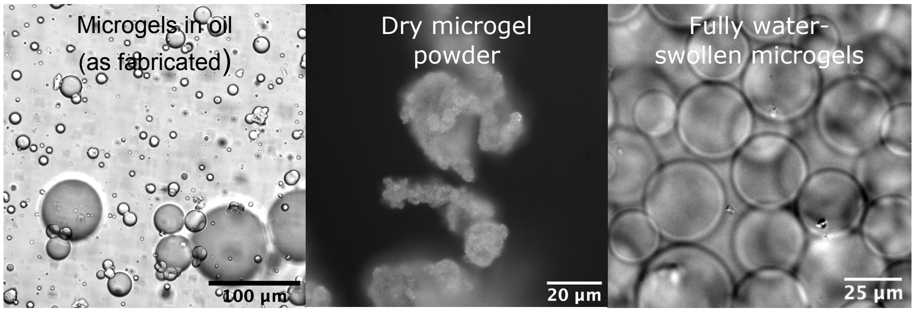

Figure 1. NaPAA microgel particles at different stages of prepataion. Left: microgels in mineral oil after emulsion polymerisation. Center: dried microgel powder consists of clumps of dried particles. Right: fully swollen microgels in de-ionized water.

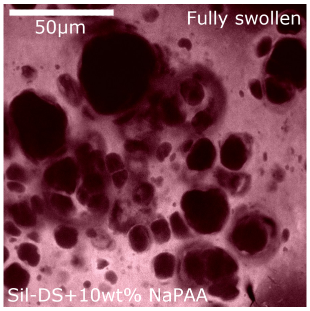

Figure 2. A confocal microscopy image of a fully swollen Sil-DS composite made originally with 10wt% dry NaPAA. For reference, the image in Figure 1 of the main paper shows a swollen sample with 30wt% dry NaPAA.

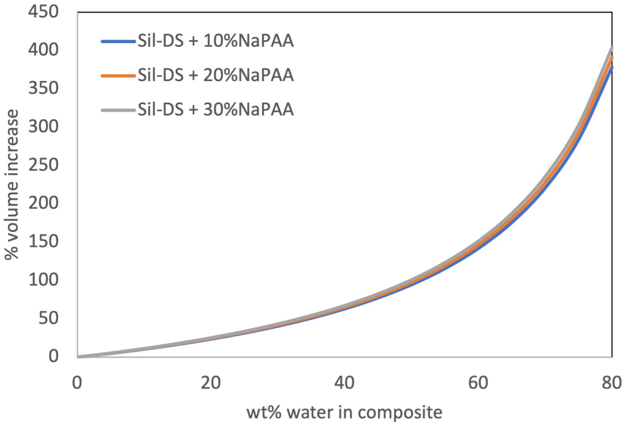

Figure 3. The relationship between the wt% of water in the samples, ϕ_w (as reported in the main text) and their % volume increase.

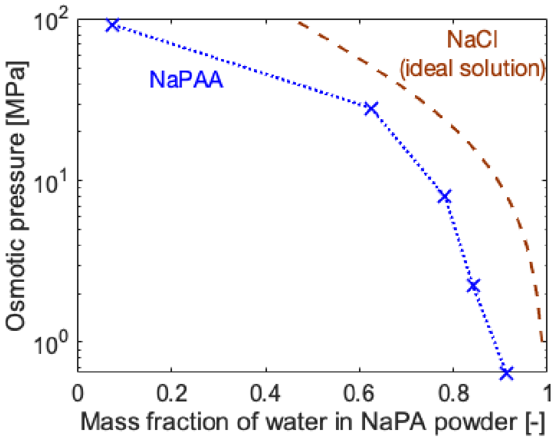

Figure 4. The osmotic pressure vs. swelling for NaPAA powder. The dashed line shows the equivalent osmotic pressure for an ideal solution of NaCl, calculated with ideal solution theory.

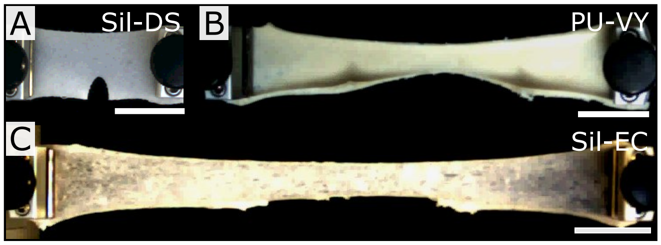

Figure 5. (A-C) Images showing notched samples of different composites at the point of failure due to mode I fracture. The samples are made with 10 wt% of dry NaPAA and are swollen to $\phi_w = 30, 51, 60$ wt% respectively. All scalebars are 2.5cm long.

V. CALCULATION OF THE INCREMENTAL MATRIX STIFFNESS

To simplify the problem we reduce the composite to a single particle growing inside an sphere of matrix material. Initially, the matrix is taken as a sphere of material with radius, R_1, with a hole in the middle of radius R_0, and has volume $V_0 = 4\pi(R_1^3 - R_0^3)/3$ (see Figure 6). The hole represents the position of a NaPAA particle. Thus, the initial volume fraction of dry NaPAA in the composite is R_0^3/R_1^3. The matrix material is modeled as a nonlinear elastic Yeoh material with strain energy den-

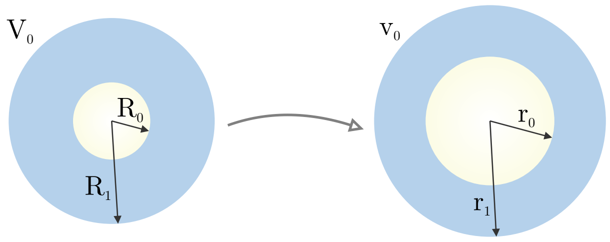

Figure 6. Inflating spherical cavity within a matrix of finite thickness. Left: Undeformed configuration, right: deformed configuration.

sity

$$W^Y = \frac{\mu}{2}\left(\mathrm{tr}(\mathbf{C}) - 3\right) + C_2\left(\mathrm{tr}(\mathbf{C}) - 3\right)^2 - \Lambda(R)(J-1). \tag{1}$$

Here, $\mathbf{C} = \mathbf{F}^T\mathbf{F}$ is the right Cauchy-Green deformation tensor, μ is the shear modulus, $J = \det(\mathbf{F})$, and $\Lambda(R)$ enforces the incompressibility constraint. Material parameters are $\mu = 0.37\,\mathrm{MPa}$ and $C_2 = 0.0186\,\mathrm{MPa}$.

As the particle expands, due to the spherical symmetry of the problem, we have $\mathbf{F} = ((\lambda_R, 0, 0), (0, \lambda_R^{-1/2}, 0), (0, 0, \lambda_R^{-1/2}))$ with

$$\lambda_R = \frac{\partial r}{\partial R} = \frac{R^2}{(r_0^3 - R_0^3 + R^3)^{2/3}}. \tag{2}$$

Here, R is the radial distance of a piece of the matrix material from the centre of the particle in the undeformed configuration, and r is the equivalent radius in the deformed configuration. We solve for $\Lambda(R)$ using a linear momentum balance and subsequently linearize the elastic modulus

$$C_{ijkl} = \frac{1}{J} F_{jJ} F_{lL} \frac{\partial^2 W^Y}{\partial F_{iJ} \partial F_{kL}}. \tag{3}$$

This expression is evaluated at each point in the material in the swollen configuration. Moving to a Cartesian frame of reference and integrating over the volume of material in the undeformed configuration gives an upper bound on the incremental stiffness tensor $\tilde{C}^{\mathrm{eff}}_{ijkl}$ of the matrix material. The stiffness in response to an applied uniaxial loading then gives the incremental unidirectional Young's modulus E_m.

Note that, to solve for E_m, there is an additional internal incompressibility constraint, $\tilde{\Lambda}$, that we must apply to ensure that the linearized swollen material does not change volume during the application of a uniaxial stretch. We solve for this using

$$\sigma_{ij} = \tilde{C}^{\mathrm{eff}}_{ijkl} u_{k,l} - \tilde{\Lambda}\delta_{ij}, \tag{4}$$

with stress-free boundary conditions perpendicular to the applied stretch. The results for composites with selected different initial loadings of dry NaPAA are shown in Figure 7.

[1] R. Long and C.-Y. Hui, Fracture toughness of hydrogels: measurement and interpretation, Soft Matter **12**, 8069 (2016).

[2] J.-Y. Sun, X. Zhao, W. R. K. Illeperuma, O. Chaudhuri, K. H. Oh, D. J. Mooney, J. J. Vlassak, and Z. Suo, Highly stretchable and tough hydrogels, Nature **489**, 133 (2012).

[3] Y. Huang, D. R. King, T. L. Sun, T. Nonoyama, T. Kurokawa, T. Nakajima, and J. P. Gong, Energy-Dissipative Matrices Enable Synergistic Toughening in Fiber Reinforced Soft Composites, Advanced Functional Materials **27**, 1605350 (2017).

[4] M. Hamdan, A. O. Sharif, G. Derwish, S. Al-Aibi, and A. Altaee, Draw solutions for Forward Osmosis process: Osmotic pressure of binary and ternary aqueous solutions of magnesium chloride, sodium chloride, sucrose and maltose, Journal of Food Engineering **155**, 10 (2015).

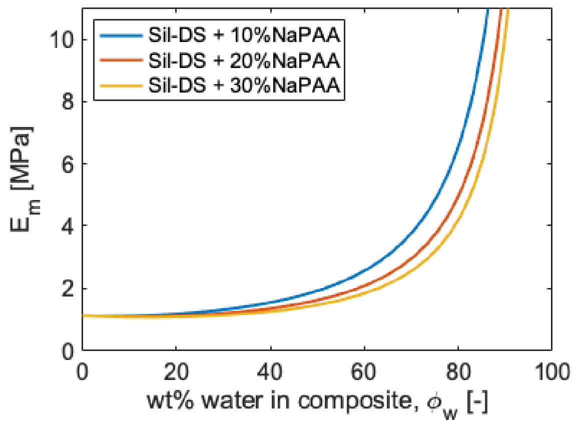

Figure 7. The effective matrix stiffness for swelling composites made with Sil-DS and NaPAA at different initial mass fractions.
